# Different Types of Intraoperative Hypotension and their Association with Post-Anesthesia Care Unit Recovery

**DOI:** 10.5334/gh.1257

**Published:** 2023-08-11

**Authors:** Lerzan Dogan, Serap Aktas Yildirim, Tugce Sarikaya, Halim Ulugol, Bulent Gucyetmez, Fevzi Toraman

**Affiliations:** 1 Department of Anesthesiology and Reanimation, Acibadem University, Istanbul, Turkey

**Keywords:** Complication, Hypotension, Intraoperative Hypotension, PACU outcomes, PACU recovery

## Abstract

**Background:**

The underlying causative mechanism leading to intraoperative hypotension (IOH) may vary depending on the stage of anesthesia and surgery, resulting in different types of IOH. Consequently, the incidence, severity, and postoperative complications associated with IOH types may differ. This study explores the association between IOH types and post-anesthesia care unit (PACU) recovery, with a focus on duration and complications.

**Methods:**

From May 2022 to December 2022, we included 4776 consecutive surgical patients aged ≥18 who underwent elective surgery with planned overnight stays at Acibadem Altunizade Hospital and received general anesthesia. Post-induction hypotension (pIOH) was defined as a decrease in blood pressure during the first 20 minutes after anesthesia induction, while maintenance intraoperative hypotension (mIOH) referred to a decrease in blood pressure occurring after the 20th minute following induction, with or without preceding pIOH.

**Results:**

Among the included patients, 22.13% experienced IOH, with a higher prevalence observed among females. Patients with mIOH exhibited higher rates of bleeding, transfusions, hypothermia, longer stays in the PACU, and increased oxygen requirements. The duration of anesthesia did not increase the likelihood of IOH. Multivariate logistic regression analysis revealed that ephedrine usage, hypothermia, the need for additional analgesics, nausea, and vomiting were factors associated with longer PACU duration. Older patients (≥65), patients with ASA≥2 status, those undergoing major surgery, experiencing unexpected bleeding, and exhibiting hypothermia at the end of anesthesia had a higher likelihood of requiring vasopressor support.

**Conclusions:**

Patients experiencing hypotension, particularly during the maintenance of anesthesia, are more prone to complications in the PACU and require closer monitoring and treatment. Although less common, mIOH has a more significant impact on outcomes compared to other factors affecting PACU recovery. The impact of mIOH on PACU duration should not be overlooked in favor of other factors.

**Registration:**

Clinicaltrials.gov identifier: NCT05671783.

## Introduction

Intraoperative hypotension (IOH) is a common occurrence during surgery. Its prevalence can vary across different regions due to various factors, such as variations in clinical practices, resources, healthcare infrastructure, and cultural differences [1]. While IOH can occur in individuals of any age, it is more commonly observed in older adults aged 65 and above [2]. There are numerous definitions of IOH in the literature [3], and the underlying causative mechanisms can vary depending on the stage of anesthesia and surgery [4]. Therefore, the incidence, severity, and recovery after different IOH types may differ.

Proper treatment response and postoperative care are crucial in managing IOH, but even with correct interventions, IOH can still occur [5]. Stabilizing the patient quickly is paramount for patient health and healthcare costs [6]. Predicting the level of postoperative care and recovery based on the type of IOH can lead to better postoperative outcomes. This study aims to determine the association between IOH types and post-anesthesia care unit (PACU) recovery, specifically looking at duration and complications in the PACU. Additionally, the study aims to compare the effect of IOH types on PACU recovery with other factors influencing PACU recovery. Our hypothesis is that different IOH types based on the stage of anesthesia are associated with different levels of postoperative follow-up and recovery in the PACU.

## Methods

This data-based observational study was conducted from May 2022 to December 2022 and included 4776 patients at Acibadem Altunizade Hospital. Ethical approval was obtained, and the study was registered with the trial registration number NCT05671783. The inclusion criteria were adult patients (≥18 years) who received general anesthesia for more than 60 minutes and underwent elective surgery with a planned overnight stay in the hospital. Patients undergoing cardiac surgery, transplant surgery, receiving vasoactive drugs prior to surgery, using mechanical circulatory support, pediatric surgery patients, and those with scheduled postoperative intensive care unit (ICU) admissions were excluded from the study. The study focused on complications during the PACU stay, and complications occurring after discharge to a regular hospital ward were not included in the analysis. The study adheres to Strengthening the Reporting of Observational Studies in Epidemiology (STROBE) guidelines. The selected articles for the literature review are relevant to the research topic and they address the key themes and issues under study.

### Data collection

Data collection included relevant variables that could influence the occurrence of IOH, such as demographic data, underlying comorbidities (hypertension, diabetes mellitus, and congestive heart failure), age, gender, and American Society of Anesthesiologists (ASA) classification. Moreover, anesthesia duration, surgical procedure severity, use of ephedrine as a vasopressor, bleeding and transfusion rates, and instances of cardiac rhythm abnormalities were intraoperatively recorded. Postoperatively, the duration in the PACU and observed complications were recorded.

### Definitions

In this study, IOH was defined as a decrease in mean blood pressure of 30% from baseline for a minimum of 5 minutes during anesthesia induction and maintenance. Post-induction hypotension (pIOH) refers to a decrease in blood pressure during the first 20 minutes after anesthesia induction [4], while maintenance intraoperative hypotension (mIOH) occurs after the 20th minute following induction. The method of measuring blood pressure (noninvasive or invasive) depended on the invasiveness of the surgical intervention and the patient’s cardiac performance.

### Anesthesia protocol

Although anesthesia management was not standardized for the study, the general anesthetic protocol followed the administration of propofol, remifentanil, and rocuronium for induction. Sevoflurane and remifentanil infusion were used to maintain anesthesia. The ventilation strategy employed a fraction of inspired oxygen (FiO2) of 40%, tidal volumes of 6–8 ml/kg, positive end-expiratory pressure (PEEP) of 3–5 mm Hg, and a respiratory rate of 12–14 breaths per minute. The definitions of intraoperative hypotension (IOH) used in this study were specific to its objectives and may not necessarily align with routine clinical practice. In daily practice, efforts are made to maintain the mean arterial pressure (MAP) above 65 mmHg to mitigate any potential negative consequences. Ephedrine was administered in cases of refractory hypotension, and if necessary, noradrenaline infusions were given. Peripheral nerve blocks or epidural anesthesia were applied when indicated, and pain management was provided with paracetamol or intravenous opioids. In the PACU, pain management continued based on individual patient needs.

### Post-anesthesia care unit

The duration of a patient’s stay in the PACU is not standardized. For the purposes of this study, we defined a long PACU duration as exceeding 30 minutes, while a short PACU duration was considered less than 30 minutes. In the PACU, the follow-up protocol involved immediate monitoring by responsible nurses upon the patient’s arrival in the PACU. If the in-room air oxygen saturation (SO2) dropped below 94%, oxygen treatment was initiated using a nasal cannula at a rate of 4 liters per minute. Clinical observation was conducted to detect respiratory and gastrointestinal complications, and antiemetics were administered in cases of nausea. Our goal is maintaining normothermia during the surgery. However, if there was a difficulty doing so, we initiated warming measures. Anesthesiologists oversaw all procedures and interventions. The Modified Aldrete Score was utilized to determine patient discharge readiness.

### Statistical analysis

Data analysis and the statistical plan were written after the data were accessed. SPSS Version 23.0 was used to conduct statistical analyses (IBM Corp., Armonk, NY, USA). According to the distribution of the values, data are presented as means, medians, and interquartile ranges (IQRs). The Kolmogorov-Smirnov test was employed to determine whether the distribution was normal. We employed student t, chi-square, and Mann-Whitney U tests for both groups’ analyses. In a multivariate logistic regression model predicting the duration in PACU, we added all significantly different parameters in the longer PACU duration group. A p-value of less than 0.05 was used to determine statistical significance. The estimated power of this study was detected as 0.75 (as per groups’ sizes [2330 and 2446], proportions for mIOH and/or pIOH [%4.4 ve %6.1] and α = 0.05).

## Results

Intraoperative hypotension (IOH) was observed in 1057 (22.13%) of the 4776 patients included in the study (Table 1), with the majority being female (2885; 60.4%). General surgery accounted for the largest proportion (1267; 26.5%) in terms of surgical specialties, followed by gynecology (861; 18.0%) and orthopedics (785; 16.4%).

**Table 1:** Patients’ characteristics and types of anesthesia and surgery.


PATIENTS, N	4776

Age, years (median)	41 (32–56)

≤40	2358 (49.4)

40> and ≤65	1778 (37.2)

>65	640 (13.4)

Sex, male, n (%)	1891 (39.6)

ASA score, (median)	1 (1–2)

ASA≥2	2120 (44.4)

Comorbidities, n (%)	

Hypertension	523 (11.0)

Diabetes mellitus	308 (6.4)

Heart failure	54 (1.1)

Type of surgery, n (%)	

General surgery	1267 (26.5)

Gynecology and obstetrics	861 (18.0)

Orthopedic	785 (16.4)

Neurosurgery	554 (11.6)

ENT surgery	502 (10.5)

Urology	389 (8.2)

Plastic surgery	253 (5.3)

Chest surgery	133 (2.8)

Eye surgery	32 (0.6)

Major surgery, n (%)	1230 (25.8)


ASA, American Society of Anesthesiology; ENT, ear, nose, and throat.

### Intraoperative hypotension (Table 2)

**Table 2:** Comparisons between intraoperative normotensive and hypotensive patients.


	NORMOTENSIVE PATIENTS (N = 3719)	IOH		

		TOTAL (N = 1057)	pIOH (N = 807)	mIOH (N = 250)

DEMOGRAPHIC DATA AND COMORBIDITIES, N (%)				

Age				

≤40	1869 (50.2)	489 (46.3)	386 (47.8)	**103 (41.2)^*ΩΩ*^**

40> and ≤65	1368 (36.8)	410 (38.8)	308 (38.2)	102 (40.8)

>65	482 (13.0)	158 (14.9)	113(14.0)	**45 (18.0)M^*ΩΩ*^**

ASA≥2	1539 (41.4)	581 (55.0)***	404 (50.1)**^*###*^**	**177 (70.8)^*ΩΩΩ, ßßß*^**

Male	1457 (39.2)	434 (41.1)	337 (41.8)	97 (38.8)

Hypertension	397 (10.7)	126 (11.9)	95 (11.8)	31 (12.4)

Diabetes mellitus	238 (6.4)	70 (6.6)	49 (6.1)	21 (8.4)

Congestive heart failure	42 (1.1)	12 (1.1)	11 (1.4)	1 (0.4)

**INTRAOPERATIVE PERIOD, N (%)**				

MAP, mmHg				

Pre-Anesthesia induction	91 (83-100)	100 (92-110)***	102 (95-110)^***###***^	**93 (85-102)^*Ω, ßßß*^**

Post-Anesthesia induction	76 (69-85)	66 (60-72)***	65 (60-71)^***###***^	**68 (61-77)^*ΩΩΩ, ßßß*^**

Major surgery	976 (26.2)	254 (24.0)	175 (21.7)^***##***^	**79 (31.6)^*ßß*^**

Ephedrin usage	396 (10.6)	415 (39.3)***	181 (22.4)^***###***^	**234 (93.6)^*ΩΩΩ, ßßß*^**

Unplanned transfusion	6 (0.2)	4 (0.4)	0 (0.0)	**4 (1.6)^*ΩΩ, ßß*^**

Unexpected bleeding	15 (0.4)	9 (0.9)	1 (0.1)	**8 (3.2)^*ΩΩΩ, ßßß*^**

Bradycardia	47 (1.3)	39 (3.7)***	22 (2.7)^***##***^	**17 (6.8)^*ΩΩΩ, ßß*^**

Tachycardia	31 (0.8)	15 (1.4)	9 (1.1)	6 (2.4)^***Ω***^

Hypothermia	43 (1.2)	29 (2.7)***	13 (1.6)	**16 (6.4)^*ΩΩΩ, ßßß*^**

**POSTOPERATIVE COMPLICATIONS, N (%)**				

Longer PACU duration	1896 (51.0)	550 (52.0)	402 (49.8)	**148 (59.2)^*Ω, ßß*^**

Postoperative SpO_2_<%90	51 (1.4)	15 (1.4)	11 (1.4)	4 (1.6)

Need for O_2_ therapy	77 (2.1)	27 (2.6)	16 (2.0)	**11 (4.4)^*Ω, ß*^**

Arrhythmia	24 (0.6)	9 (0.9)	7 (0.9)	2 (0.8)

Bradycardia or Tachycardia	32 (0.9)	17 (1.6)*	13 (1.6)	4 (1.6)

Nausea	193 (5.2)	51 (4.8)	34 (4.2)	17 (6.8)

Vomiting	57 (1.5)	20 (1.9)	12 (1.5)	8 (3.2)^***Ω***^

Unplanned ICU admission	2 (0.1)	6 (0.6)**	4 (0.5)^***#***^	2 (0.8)^***Ω***^


ASA, American Society of Anesthesiology; mIOH, maintenance intraoperative hypotension; O_2_, Oxygen; PACU, Post Anesthesia Care Unit; pIOH, Post-induction hypotension. *; *comparison between normotensive and total hypotensive patients*, ^#^; *comparison between normotensive and pIOH patients*, ^Ω^; *comparison between normotensive and mIOH patients*, β; comparison between pIOH and mIOH patients. * and ^#^ and ^Ω^ and ^ß^, p; 0.05–0.01. ** and ^##^ and ^ΩΩ^ and ^ßß^, p; 0.01–0.001. *** and ^###^ and ^ΩΩΩ^ and ^ßßß^, p < 0.001.

Except for age over 65 and higher ASA scores, no correlations were found between the development of IOH and gender, comorbidities, or complications that may arise in the PACU. However, the incidence of complications varied among IOH types depending on the phase of anesthesia during which it occurred. The pIOH group showed a lower association with complications compared to other groups. In contrast, patients with mIOH demonstrated a higher rate of bleeding, necessitated more transfusions, and had a higher prevalence of hypothermia at the end of anesthesia. Postoperatively, the mIOH group exhibited a longer PACU stay and a greater requirement for oxygen (O2) in the PACU. When all the factors affecting PACU were evaluated, mIOH remained statistically significant. Another notable finding was that a longer duration in the PACU remained significant only in the mIOH group, while unplanned intensive care unit (ICU) admission was significant in both IOH types (Table 4).

### Vasopressor usage (Table 3)

**Table 3:** Comparisons between patients with ephedrine (–) and (+) in intraoperative period.


	EPHEDRINE (–) (N = 3965)	EPHEDRINE (+) (N = 811)	P

DEMOGRAPHIC DATA AND COMORBIDITIES, N (%)			

Age			***<0.001***

≤40	2016 (50.9)	342 (42.2)	

40> and ≤65	1448 (36.5)	330 (40.7)	

>65	501(12.6)	136 (17.1)	

ASA≥2	1594 (40.2)	526 (64.9)	***<0.001***

Male	1555 (39.2)	336 (41.4)	0.241

Hypertension	416 (10.5)	107 (13.2)	***0.025***

Diabetes mellitus	247 (6.2)	61 (7.5)	0.172

Congestive heart failure	44 (1.1)	10 (1.2)	0.762

**INTRAOPERATIVE PERIOD, N (%)**			

Major surgery	997 (25.1)	233 (28.7)	***0.033***

pIOH	629 (15.9)	263 (32.4)	***<0.001***

mIOH and/or pIOH	16 (0.4)	234 (28.9)	***<0.001***

Bradycardia	48 (1.2)	38 (4.7)	***<0.001***

Tachycardia	33 (0.8)	13 (1.6)	***0.041***

Unplanned transfusion	6 (0.2)	4 (0.5)	0.074

Unexpected bleeding	12 (0.3)	12 (1.5)	***<0.001***

Hypotermia at the end of the surgery	39 (1.0)	33 (4.1)	***<0.001***

**POSTOPERATIVE COMPLICATIONS, N (%)**			

Duration of PACU >30min	1973 (49.8)	473 (58.3)	***<0.001***

Postoperative SpO_2_<%90	50 (1.3)	16 (2.0)	0.114

Need for O_2_ therapy	70 (1.8)	34 (4.2)	***<0.001***

Arrhythmia	28 (0.7)	5 (0.6)	0.779

Bradycardia or Tachycardia	33 (0.8)	16 (2.0)	***0.003***

Nausea	198 (5.0)	46 (5.7)	0.424

Vomiting	61 (1.5)	16 (2.0)	0.371

Unplanned ICU admission	4 (0.1)	4 (0.5)	***0.033***


ASA, American Society of Anesthesiology; mIOH, maintenance intraoperative hypotension; O_2_, Oxygen; PACU, Post Anesthesia Care Unit; SpO_2_, oxygen saturation; pIOH, Post-induction hypotension.

Among patients who received ephedrine, a higher proportion of them were older than 65 years (p = 0.029) and had ASA≥2 status (p < 0.001). They also underwent major surgery (p = 0.033) and experienced more cases of unexpected bleeding (p < 0.001) and hypothermia at the end of anesthesia (p < 0.001). Additionally, these patients required more oxygen in the PACU (p < 0.001). While IOH groups were only observed for intraoperative heart rate variability, patients who received ephedrine were monitored for multiple complications, although no significant arrhythmias were observed (p = 0.779). Furthermore, the use of ephedrine was associated with a significantly higher rate of unplanned ICU admission.

### All factors affecting PACU duration (Table 4)

**Table 4:** Factors affecting the length of stay in the PACU.


	SHORT PACU DURATION (N = 2330)	LONG PACU DURATION (N = 2446)	P

PREOPERATIVE PERIOD, N (%)			

Age			0.618

≤40	1193 (48.8)	1165 (50.0)	

40> and ≤65	916 (37.4)	862 (37.0)	

>65	337 (13.8)	385 (15.7)	

ASA≥2	985 (42.3)	1135 (46.4)	***0.004***

Male	952 (40.9)	939 (38.4)	0.081

Hypertension	255 (10.9)	268 (11.0)	0.989

Diabetes mellitus	149 (6.4)	159 (6.5)	0.882

Congestive heart failure	26 (1.1)	28 (1.1)	0.925

Preoperative SpO2<%95	41 (1.8)	58 (2.4)	0.138

**INTRAOPERATIVE PERIOD, N (%)**			

Major surgery, n (%)	560 (24.0)	670 (27.4)	***0.008***

pIOH	445 (19.1)	447 (18.3)	0.465

mIOH	102 (4.4)	148 (6.1)	***0.009***

Bradycardia	45 (1.9)	41 (1.7)	0.507

Tachycardia	17 (0.7)	29 (1.2)	0.107

Ephedrine usage	338 (14.5)	473 (19.3)	***<0.001***

Unexpected bleeding	6 (0.3)	18 (0.7)	***0.019***

Unscheduled transfusion	1 (0.1)	9 (0.4)	***0.013***

Hypotermia	20 (0.9)	52 (2.1)	***<0.001***

**PACU PERIOD, N (%)**			

Nausea	49 (2.1)	195 (8.0)	***<0.001***

Vomiting	12 (0.5)	65 (2.7)	***<0.001***

Need for O_2_ therapy (>4L min^-1^)	42 (1.8)	62 (2.5)	0.083

Need for additional analgesics	90 (3.9)	731 (29.9)	***<0.001***


ASA, American Society of Anesthesiology; mIOH, maintenance intraoperative hypotension; O_2_, Oxygen; PACU, Post Anesthesia Care Unit; SpO_2_, oxygen saturation; pIOH, Post-induction hypotension.

Preoperative data revealed that only ASA≥2 status (p < 0.001) was significantly associated with a longer PACU duration. Major surgery (p = 0.008), mIOH (p = 0.009), ephedrine use (p = 0.001), unexpected bleeding (p = 0.019), unscheduled transfusion (p = 0.013), and hypothermia (p = 0.001) were also associated with prolonged PACU duration. Notably, pIOH (p = 0.465) did not affect PACU duration, while mIOH (p = 0.009) did. Postoperative factors that significantly increased PACU duration included nausea (p < 0.001), vomiting (p < 0.001), the need for additional analgesics (p < 0.001), and the requirement for emergency intervention (p = 0.003). A multivariate logistic regression analysis was performed to identify the factors contributing to this relationship. The analysis revealed that ephedrine usage (OR = 1.3; 95% CI: 1.1–1.6), hypothermia at the end of anesthesia (OR = 2.1; 95% CI: 1.2–3.6), the need for additional analgesics during the postoperative period (OR = 10.3; 95% CI: 8.2–13.0), nausea (OR = 3.1; 95% CI: 2.1–4.4), and vomiting (OR = 2.1; 95% CI: 1.1–4.2) were all significantly associated with a longer duration in the PACU (Table 5).

**Table 5:** Multivariate logistic regression model for the likelihood of the PACU duration>30 min.


	OR (CI 95%)	P

PREOPERATIVE PERIOD		

ASA≥2	1.1 (0.9–1.2)	0.153

**INTRAOPERATIVE PERIOD**		

Ephedrine usage	1.3 (1.1–1.6)	***0.003***

Hypothermia at the end of the surgery	2.1 (1.2–3.6)	***0.010***

Unexpected bleeding	2.4 (0.9–6.4)	0.082

Major surgery	1.1 (0.9–1.3)	0.102

Unplanned transfusion	5.7 (0.7–48.2)	0.113

mIOH	0.9 (0.7–1.4)	0.897

**PACU PERIOD**		

Need for additional analgesics	10.3 (8.2-13.0)	***<0.001***

Nausea	3.1 (2.1-4.4)	***<0.001***

Vomiting	2.1 (1.1-4.2)	***0.037***


ASA, American Society of Anesthesiology; mIOH, maintenance intraoperative hypotension; O_2_, Oxygen; PACU, Post Anesthesia Care Unit; SpO_2_, oxygen saturation; pIOH, Post-induction hypotension.

## Discussion

The present observational study aimed to explore the occurrence of different types of intraoperative hypotension and their associated complications in the post-anesthesia care unit. Our findings demonstrate that different types of hypotension are linked to varying durations in the PACU and complication rates (Table 2). The underlying mechanisms and phases of IOH contribute to these differences [4], leading to distinct challenges in treatment and associated complications. Post-induction hypotension was more prevalent than hypotension during anesthesia maintenance, consistent with previous studies [3], but it did not impact PACU duration or complications. On the other hand, mIOH was less common but had a significant impact on PACU duration and related complications. The underlying causes of hypotension during different stages of anesthesia and surgery explain this pattern.

Immediately after the induction of general anesthesia, regardless of the type of surgery, hypotension can occur due to the inhibition of heart function and vasodilation caused by anesthetic agents [7]. During this post-induction period, the absence of surgical stimuli and distractions for anesthesia adjustments [8] may lead to delayed recognition and management of pIOH, resulting in negative outcomes [9]. Hypotension during pIOH period is due to the inhibition of heart function and vasodilatation by anesthetics and the interplay between them [10]. In contrast, hypotension during the maintenance of anesthesia is due to the nature and course of the surgical procedure, and its treatment is more difficult [11]. Factors like bleeding, relative hypovolemia, vasodilation caused by anesthetic side effects, neuraxial anesthesia, and systemic inflammation can contribute to mIOH [11]. These underlying mechanisms make mIOH more challenging to treat and are associated with a higher incidence of complications, emphasizing the need for stricter management.

When discussing IOH, it is important to recognize hypovolemia as of the most common causes [12]. Optimizing fluid therapy preoperative or during surgery may not always be feasible, and a gradual approach has been associated with better outcomes [13]. Moreover, fluid therapy alone may not suffice for hypotension caused by vasodilation, necessitating the use of vasopressors to maintain adequate blood pressure [11,14]. A prompt response to hypotension is crucial, as sustained uncorrected hypotension can lead to irreversible ischemic damage by impairing organ perfusion [15]. Confirmation bias during crises has been identified as a reason for incorrect diagnoses when hypotension persists at dangerous levels [16]. Regardless of the underlying cause, prompt review of correctable factors and timely initiation of vasopressor treatment are crucial [17]. In our study, ephedrine was frequently used in both hypotension groups, with a high overall usage rate (17.0%; n = 811). It is important to note that the definition used in our study was specific to the research purpose, while in practice, ephedrine was administered immediately when non-vasopressor regimens failed. Contrary to previous findings [18], the administration of ephedrine in our study did not result in postoperative arrhythmias. Some patients in the ephedrine usage group exhibited significant tachycardia, which can be considered a compensatory mechanism, especially in cases of intraoperative bleeding. Additionally, the discussion about the relative risks of hypoperfusion versus vasopressor use clearly favors the prompt administration of vasopressors, as the negative effects are primarily observed with prolonged use, particularly in critically ill patients [19].

The individual variability in compensatory mechanisms contributes to the severity and recovery from hypotension [3]. Therefore, individualized management strategies should be considered, particularly for patients with limited cardiac reserve [15]. Their management involves preoperative optimizing cardiac function, closely monitoring hemodynamics, titrating anesthetics carefully, maintaining euvolemia, and avoiding volume overload, all tailored to the individual’s condition to minimize perioperative risks [20,21]. Consistent with previous research [22], our results demonstrated that older patients and those with higher ASA scores were susceptible to intraoperative hypotension. However, in our study, patients with hypertension, diabetes, or congestive heart failure did not show an association with PACU complications, although the follow-up duration may not have been sufficient to detect the adverse outcomes reported in the literature. It is crucial to acknowledge that these patients should not be assumed to be entirely free of complications. Our study sought to examine the outcomes of IOH types in routine clinical practice. These patients’ heightened vulnerability warranted an elevated level of sensitivity and effort in their management.

The identification of risk factors for perioperative complications and adequate optimization are integral to anesthetic management [23]. However, in cases where non-modifiable factors such as age and ASA score are present, complete optimization may not always be possible [24]. Modifiable factors, such as close intraoperative monitoring and prompt interventions, can influence postoperative complications [1]. Our results support previous studies that have identified risk factors for PACU duration and complications [25]. Factors such as mIOH, treatment with ephedrine, unscheduled transfusion due to unexpected bleeding, and hypothermia at the end of the surgery were associated with increased PACU duration and complications (Table 4). Notably, mIOH had a significant impact on PACU recovery, while pIOH had a relatively benign effect due to the influence of anesthetics. Both IOH groups and the ephedrine usage group exhibited an increased need for unplanned ICU admission, emphasizing the importance of proper communication between the operating room and the ICU [1]. However, the question of why hypotension affects postoperative recovery remains. Despite advancements in the understanding of hypotension, the restoration of normotension remains a challenge. There is a possibility that transient breakdowns of autonomic regulatory mechanisms influence the recovery from intraoperative hypotension. Cerebral hipoperfusion has been shown to impair cerebral and autonomic regulation [26], and even controlled hypotensive anesthesia has been associated with cases of hypoperfusion. Additionally, complications may be related to the severity and duration of hypotension [27]. However, we lack evidence on whether short-term decreases in blood pressure below a certain threshold impact regulatory systems. In the multivariate logistic regression model for PACU duration, pIOH lost its significance, while hypothermia and ephedrine usage maintained their significance.

Our study has several limitations. Firstly, although our deep hypotensive events did not exceed five minutes, we do not have precise information regarding their exact degree and duration. Previous studies have shown that the time spent with hypotension independently increases the risk of adverse outcomes [28,29]. Secondly, we did not include central nervous system findings, but this may not be considered a limitation. Central nervous system changes may go undetected without preoperative cognitive evaluation [30], and the role of intraoperative hypotension in the development of postoperative cognitive dysfunction remains unclear [31]. Nevertheless, we believe that these aspects should be thoroughly evaluated in separate subject-oriented studies. Furthermore, several future expectations can be inferred that may influence medical conduct regarding IOH. These include studying hypoperfusion and vasopressor-related complications, the impact of short-term blood pressure decreases on regulatory systems, and the long-term consequences of autonomic regulatory mechanisms during hypotension on postoperative recovery and outcomes.

These expectations include an evaluation of hypotension’s impact on postoperative cognitive dysfunction, and further investigation of hypoperfusion and vasopressor-related complications. Research is also needed to evaluate the impact of short-term blood pressure decreases below a certain threshold on regulatory systems. Understanding whether transient breakdowns of autonomic regulatory mechanisms during hypotension have long-term consequences on postoperative recovery and outcomes will provide insights to refine interventions and patient management strategies.

## Conclusion

Patients experiencing hypotension, particularly during the maintenance of anesthesia, are more prone to complications in the PACU and require closer monitoring and treatment. Although less common, mIOH has a more significant impact on outcomes compared to other factors affecting PACU recovery. The impact of mIOH on PACU duration should not be overlooked in favor of other factors.

## Data Accessibility Statements

Data is available upon request.
